# 643. Cefiderocol *In vitro* Activity Against Molecularly Characterized *Pseudomonas aeruginosa* and *Acinetobacter baumannii-calcoaceticus* complex Clinical Isolates Causing Infection in United States Hospitals (2020-2021)

**DOI:** 10.1093/ofid/ofac492.695

**Published:** 2022-12-15

**Authors:** Rodrigo E Mendes, Cory Hubler, Valerie Kantro, Dee Shortridge, Helio S Sader, Jennifer M Streit, Mariana Castanheira

**Affiliations:** JMI Laboratories, North Liberty, Iowa; JMI Laboratories, North Liberty, Iowa; JMI Laboratories, North Liberty, Iowa; JMI Laboratories, North Liberty, Iowa; JMI Laboratories, North Liberty, Iowa; JMI Laboratories, North Liberty, Iowa; JMI Laboratories, North Liberty, Iowa

## Abstract

**Background:**

Cefiderocol (CFDC) is a siderophore-conjugated cephalosporin with activity against Gram-negative bacteria. CFDC and comparator activities were analyzed against resistant and molecularly characterized *P. aeruginosa* (PSA) and *A. baumannii-calcoaceticus* complex (ACB) as part of the SENTRY Antimicrobial Surveillance Program.

**Figure:**
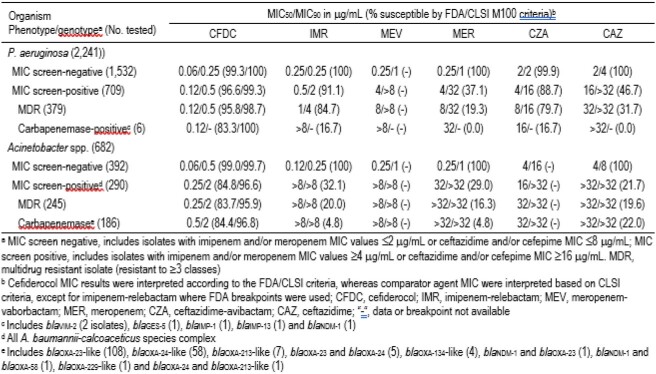

**Methods:**

2,241 PSA and 682 ACB were consecutively collected from 63 US sites in 2020-2021. Susceptibility testing was performed by broth microdilution and CFDC testing used iron-depleted media. FDA and CLSI breakpoints were used for CFDC. CLSI criteria were applied to comparators, except for imipenem-relebactam (IMR) that used FDA breakpoints. Isolates with a resistance phenotype to ≥ 3 classes were defined as multidrug resistant (MDR). ACB and PSA with imipenem and/or meropenem (MER) MIC ≥4 μg/mL or ceftazidime (CAZ) and/or cefepime MIC ≥ 16 μg/mL were subjected to next-generation genome sequencing for screening of acquired carbapenemase genes.

**Results:**

31.6% (709/2,241) and 16.9% (379/2,241) of PSA met the MIC screening criteria and showed an MDR phenotype, respectively (Table). Carbapenemase genes were detected in 6 (< 1%) PSA. CFDC had similar MIC against PSA that did not (MIC_50/90_, 0.06/0.25 μg/mL) and did (MIC_50/90_, 0.12/0.5 μg/mL) meet the MIC screening criteria. CFDC also had similar MIC_50_ values (0.12 μg/mL) against the MDR and carbapenemase-positive PSA populations, whereas other agents had compromised activity. 42.5% (290/682) of ACB met the MIC screening criteria, while 35.9% (245/682) had an MDR phenotype, and 27.3% carried carbapenemase genes (all OXA carbapenemase, except for 2 *bla*_NDM-1_). In general, CFDC, IMR, meropenem-vaborbactam, MER, ceftazidime-avibactam, and CAZ had activity against ACB that did not meet the MIC screening criteria, but only CFDC (MIC_50/90_, 0.25-0.5/2 μg/mL) was active against the resistant ACB subsets.

**Conclusion:**

Many PSA showed a resistance phenotype but acquired carbapenemase genes remained rare in this subset. In contrast, resistance and presence of carbapenemase genes were high in ACB. CFDC showed potent activity against PSA and ACB subsets in US hospitals, including across resistant and molecularly characterized subsets, where treatment options were limited.

**Disclosures:**

**Rodrigo E. Mendes, PhD**, AbbVie: Grant/Research Support|Cidara: Grant/Research Support|GSK: Grant/Research Support|Melinta: Grant/Research Support|Nabriva Therapeutics: Grant/Research Support|Office for Assistant Secretary of Defense for Health Affairs: Grant/Research Support|Pfizer: Grant/Research Support|Shionogi: Grant/Research Support|Spero Therapeutics: Grant/Research Support **Cory Hubler, BS**, Melinta: Grant/Research Support|Shionogi: Grant/Research Support **Valerie Kantro, BA**, AbbVie: Grant/Research Support|GSK: Grant/Research Support|Melinta: Grant/Research Support|Shionogi: Grant/Research Support **Dee Shortridge, PhD**, AbbVie: Grant/Research Support|JMI Laboratory: Employee|Melinta: Grant/Research Support|Menarini: Grant/Research Support|Shionogi: Grant/Research Support **Helio S. Sader, MD, PhD**, AbbVie: Grant/Research Support|Cidara: Grant/Research Support|Melinta: Grant/Research Support|Nabriva Therapeutics: Grant/Research Support|Pfizer: Grant/Research Support **Jennifer M. Streit, BS, MT(ASCP)**, Cidara: Grant/Research Support|GSK: Grant/Research Support|Melinta: Grant/Research Support|Shionogi: Grant/Research Support **Mariana Castanheira, PhD**, AbbVie: Grant/Research Support|Cidara: Grant/Research Support|GSK: Grant/Research Support|Melinta: Grant/Research Support|Pfizer: Grant/Research Support|Shionogi: Grant/Research Support.

